# Comparative Studies on Synthesis, Characterization and Photocatalytic Activity of Ag Doped ZnO Nanoparticles

**DOI:** 10.1021/acsomega.2c07499

**Published:** 2023-02-13

**Authors:** Snehal S. Wagh, Vishal S. Kadam, Chaitali V. Jagtap, Dipak B Salunkhe, Rajendra S. Patil, Habib M. Pathan, Shashikant P. Patole

**Affiliations:** † School of Polytechnic and Skill Development, Dr. Vishwanath Karad MIT World Peace University, Pune, 411038, India; ‡ Advanced Physics Laboratory, Department of Physics, Savitribai Phule Pune University, Pune, 411007, India; § PSGVPM ASC College, Shahada, Nandurbar 425409, India; ∥ Kisan ASC College, Parola, Jalgaon 425111, India; ⊥ Department of Physics, Khalifa University of Science and Technology, Abu Dhabi, 127788, United Arab Emirates

## Abstract

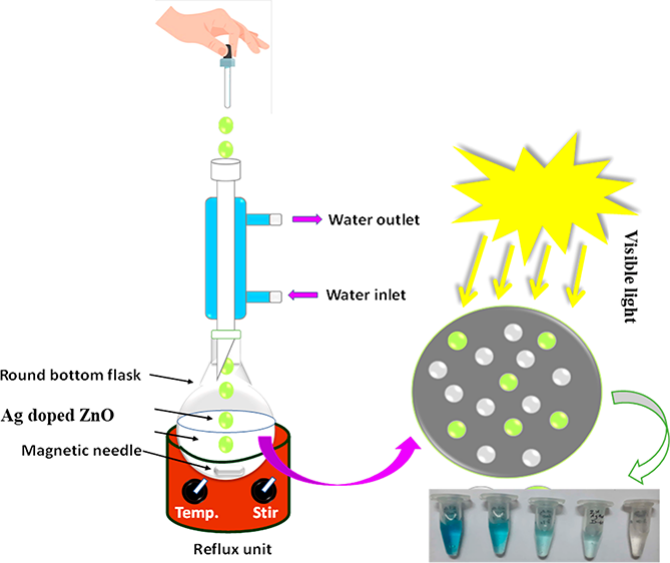

In this work, silver (Ag) doped zinc oxide (ZnO) nanoparticles were synthesized using zinc chloride, zinc nitrate, and zinc acetate precursors with (0 to 10) wt % Ag doping by a simple reflux chemical method. The nanoparticles were characterized by X-ray diffraction, scanning electron microscopy, transmission electron microscopy, ultraviolet visible spectroscopy, and photoluminescence spectroscopy. The nanoparticles are studied as a photocatalyst for visible light driven annihilation of methylene blue and rose bengal dyes. The 5 wt % Ag doped ZnO displayed optimum photocatalytic activity toward methylene blue and rose bengal dye degradation at the rate of 13 × 10^–2^ min^–1^ and 10 × 10^–2^ min^–1^, respectively. Here we report antifungal activity for the first time using Ag doped ZnO nanoparticles against Bipolaris sorokiniana, displaying 45% efficiency for 7 wt % Ag doped ZnO.

## 1 Introduction

The hasty widening of globalized world and industrial development has instigated severe environmental pollution complications. According to the United Nations report on the state of the world’s water, more than 5 billion people could suffer water scarcities by 2050 due to change in climatic conditions, increase in demand of water and polluted water resources. The removal of dyes and other impurities from the water bodies has become a puzzling assignment in modern times. Hence, numerous approaches have been implemented to tackle such issues.^1−3^ Photocatalysis may be the top remedy in capturing the sunlight and degrading water pollutants.^4^

Currently, the artificial dyes are widely utilized in production of goods such as colored clothes, leather decorations, furniture, and plastic products, but almost 12% of the dyes are eliminated as leftover, and ∼20% of such excess is dumped into the environment.^5^ The process of annihilation of dyes involves oxidation of hefty dye molecules into inconsequential particles such as water, carbon dioxide, and other mineral byproducts. As specified, complete usage of dye molecules in the dyeing process is not possible, which in turn gives rise to the existence of dye molecules in aqueous leftovers removed from the manufacturing units. Heterogeneous photocatalysis is one of many contemporary approaches which is broadly engaged for the degradation of the dyes.^6^ In this method, when light of proper wavelength falls on the exterior of a semiconducting material, an electron–hole pair is generated, transferring the electrons from the valence band (VB) to the conduction band (CB). These excitons respond with surface adsorbed oxygen and the water molecules, which in turn generates superoxide anion and the hydroxyl (OH) radical, respectively. These OH and superoxide radicals have the capability of reducing as well as oxidizing a large number of pollutant compounds.^6^

In previous studies, titanium dioxide (TiO_2_) nanoparticles were explored as a photocatalytic material. Yet, the TiO_2_ band gap of around 3.2 eV^7^ limits their photocatalysis activity to the ultraviolet (UV) region which involves less than 5% of the full solar energy.^8^ Along with this, the quick recombination rate of photogenerated excitons tends to reduce photocatalytic activity.^9^ Apart from TiO_2_, numerous other semiconducting arrangements like zinc sulfide (ZnS), cerium oxide (CeO_2_), magnesium oxide (MgO), cadmium sulfide (CdS), zirconium oxide (ZrO_2_), and tungsten oxide (WO_3_) have been involved in the photocatalytic degradation of dyes.^10−13^ Most of these arrangements show higher values of energy band gap, and thus UV photon sources are required to clean the wastewater, as that of TiO_2_. Zinc oxide (ZnO) is a broadly investigated material in the scientific community. ZnO was studied for its antioxidant effect and photocatalytic effect on victoria blue dye having 93% efficiency for a catalytic dose of 0.75 mg·mL^–1^.^14^ Antifungal activity was reported by Ali Es-haghi et al.^15^ Poly(ethylene glycol) (PEG) based ZnO has been reported showing induced behavioral effects in rats.^16^ The antibacterial effect of ZnO against S. aureus, *E. coli* with an efficiency of approximately 96% and photocatalytic degradation of methylene blue (efficiency- 95%) has been reported by Sujeong Kim et al.^17^

ZnO demonstrates excellent properties such as curtailed particles on the order of nanometers, crystallographic orientation, density and size, morphology, and high aspect ratio. Therefore, it is broadly considered by researchers in the pursuit of superb optical, chemical, physical, and electronic characteristics in the direction of increasing manufacturing and technical application.^18,19^ These properties demonstrate decisive characteristics in various applications like actuators, photoemitters, transducers, sensors, and catalysts. In previously reported work, the dye degradation performance of nickel and thorium codoped ZnO was studied for methylene blue (MB) dye which has shown 93% photocatalytic efficiency in 180 min at pH-10.^20^ Dysprosium doped ZnO, prepared by a combustion method with the assistance of tartaric acid, displayed a MB degradation efficiency of 97% within 75 min.^21^ Cobalt (Co) and manganese (Mn) doped ZnO, synthesized by the coprecipitation method, had shown maximum photocatalytic performance for methyl orange (MO) at 12 wt % of Mn at pH value of 4.^22^ A hybrid of magnesium (Mg) doped ZnO and reduced graphine oxide (RGO) were reported with an efficiency of 95% for MB dye in 60 min.^23^ Türkyılmaz et al. reported that Mn, Fe, Ag, Ni, and Ag doped ZnO arranged by the one step hydrothermal method to degrade tartrazine had shown the highest degradation rate of 98% in 60 min using Ni/ZnO.^24^ 4% zirconium (Zr (IV)) doped ZnO composite prepared via the sol–gel method had shown optimum photocatalytic efficiency in 40 min of irradiation time.^25^ Mg doped ZnO showed 99.6% degradation efficiency in 150 min for 4-chlorophenol.^26^ Mg/ZnO-GO composite synthesized using the wet impregnation method displayed 97% degradation efficiency in 60 min for indigo carmine dye.^27^ Reduced graphine oxide-ZnO composite was reported for removal of rhodamine B (RhB), MB dyes, and tea stain from cotton fibers under irradiation of sunlight.^28^

There are wide studies accomplished on photocatalytic behavior of silver doped zinc oxide with multiple dyes such as MB, brilliant blue, MO, rose bengal (RB), naphthol blue black, rhodamine, etc.^29−31^ Ag–Cu_2_O/ZnO nanorods prepared by a modified solvothermal method were reported for photocatalytic CO_2_ reduction.^32^ The study of the degradation mechanism of such dyes is still under progress for betterment of results. In Ag doped ZnO, silver can substitute Zn sites, and it may show its presence in interstitial sites.^33,34^ The tuned bandgap energy of ≈2.4 eV of Ag doped ZnO makes it more sensitive toward visible light irradiation along with silver showing surface plasmon resonance (SPR) effect confirming to its visible light sensitivity. Thus, Ag doped ZnO provides more opportunities than pristine ZnO toward betterment of its photocatalytic performance against organic contaminants.

In the present work, annihilation of MB and RB dyes as symbolic organic impurities under visible light irradiation were studied with (0, 2, 5, 7, and 10 wt %) Ag doped ZnO prepared via refluxed chemical method using zinc chloride (ZCL), zinc nitrate (ZN), and zinc acetate (ZAC) as cationic precursor sources to explore their dye degradation performance and to make comparative studies. The outcomes of the recorded observations reveal that the gathering of Ag onto the ZnO array considerably improves its dye degradation abilities.^35^

Along with photocatalytic activity, we have explored the antifungal characteristics of Ag doped ZnO. The spot blotch of wheat is the most critical widespread disease found in warmer areas of South Asia and the other regions where wheat is grown, resulting in grain yield loss. Bipolaris sorokiniana (Sacc.) Shoem (syn. Helminthosporium sativum, teleomorph Cochliobolous sativus) causes the spot blotch in wheat leading to the destruction of crops.^36^ To control disease, fungicides are commonly utilized to reduce the harshness of spot blotch, but these synthetic fungicides are less eco-friendly. Ag nanoparticles, zinc peroxide (ZnO_2_), and zinc nanoparticles were separately investigated against Bipolaris sorokiniana.^37−39^ Instead, the Ag doped ZnO nanoparticle is one of the promising options to reduce disease severity, as it is less harmful to plants and animals.^40^ There have been comparatively limited studies on the applications of Ag doped ZnO nanoparticles to regulate plant diseases. Hence, in the present work we have also investigated antifungal abilities of Ag doped ZnO along with its photocatalysis activity.

## 2 Experimental Section

### 2.1 Materials

In this study, as-purchased analytical grade chemicals were used in the experimentation process. Zinc chloride (Thomas Baker (Chemicals) Pvt. Ltd.) purity 98%, zinc acetate dihydrate (Sisco Research Lab (SRL)), zinc nitrate hexahydrate (Merck life sciences pvt.ltd.), diethylene glycol (Sisco Research Lab (SRL)), silver nitrate (Thomas Baker (Chemicals) Pvt. Ltd.) purity 99.8%, sodium hydroxide pellets (ACROS organics) and methylene blue (Sisco Research Lab (SRL)), rose bengal (Molychem), and potato dextrose agar medium (Himedia, Mumbai, India) were commercially purchased. In this study, all the solutions were prepared in double distilled water (DDW).

### 2.2 Synthesis of Silver Doped Zinc Oxide Nanoparticles

A sequence of Ag doped ZnO nanoparticles was prepared by adding zinc chloride, zinc nitrate, and zinc acetate in double distilled water with (0, 2, 5, 7, and 10) wt % silver nitrate. Each 1 M zinc precursor solution was liquefied in 100 mL of DDW at 100 °C under the refluxed chemical method approach for 1 h, trailed by the addition of 20 mL of diethylene glycol with constant stirring for another 1 h at 100 °C. Silver nitrate solutions were prepared in 50 mL of DDW separately and poured into above precursor solutions with magnetic stirring for the next 30 min. Then 2 M sodium hydroxide (NaOH) solution was inserted dropwise at the rate of 1 drop per second to the above prepared zinc precursor solution and kept stirring for an additional 2 h.^41^ The above prepared solution was cooled overnight, appearing brown in color, and it was further rinsed and washed multiple times and dried at 70 °C for 20 h. A fine powder was prepared by grinding this dried sample followed by annealing at 300 °C for around 2 h resulting in Ag doped ZnO nanoparticles. Thus, (0, 2, 5, 7, 10 wt %) silver doped Zinc oxide samples were synthesized with zinc chloride precursor and were labeled as ZCL-A, ZCL-B, ZCL-C, ZCL-D, and ZCL-E, respectively. Samples prepared with zinc nitrate precursor were named ZN-A, ZN-B, ZN-C, ZN-D, and ZN-E, respectively. And samples prepared with zinc acetate precursor were named ZAC-A, ZAC-B, ZAC-C, ZAC-D, and ZAC-E, respectively.

### 2.3 Preparation of Media

The antifungal performance of the Ag doped ZnO was determined using the agar diffusion method. To prepare this assay, the potato dextrose agar (PDA) medium was added in 100 mL of DDW and the solution was heated until it dissolved completely. To sterilize, the PDA and glass Petri dishes were kept in an autoclave for 30 min at a pressure of 15 Pa. The PDA medium was dispensed into the Petri dishes using a laminar flow chamber. After the medium solidified, 20 mg of the prepared samples was spread on the Petri plate. Then, a fungal disk of Bipolaris sorokiniana was inoculated at the center of the Petri dish. The potato dextrose agar disk was taken out with a sterile cork borer from the center for the inoculation. For the comparison, control was maintained without Ag-doped ZnO nanoparticles. These plates were incubated at 25 °C for 72 h. The fungal growth area was observed. The diameter of such zones of inhibition was calculated, and antifungal activity was calculated by the poisoned food method.^42^

### 2.4 Characterization Methods

The crystal structure was studied using X-ray diffraction (XRD) (D/B max-2400, Rigaku, USA) to understand phase and average crystallite size of ZnO nanoparticles. Morphology was studied using scanning electron microscopy (SEM) (JEOL JSM 6360-A, USA), and transmission electron microscopy (TEM) (FEI TECNAI G2 Spirit TWIN 120 kV). Optical study was performed using UV–vis spectrophotometer (JASCO V-670, Germany), and photoluminescence (PL) measurements were performed and recorded by PL spectrometer (Horiba Fluorolog). OSRAM 300 W halogen lamp was used as the source of visible light (emission range ∼400–800 nm) to study the photocatalytic degradation of MB and RB dyes.

## 3 Results and Discussion

### 3.1 XRD Studies

**Figure 1:**
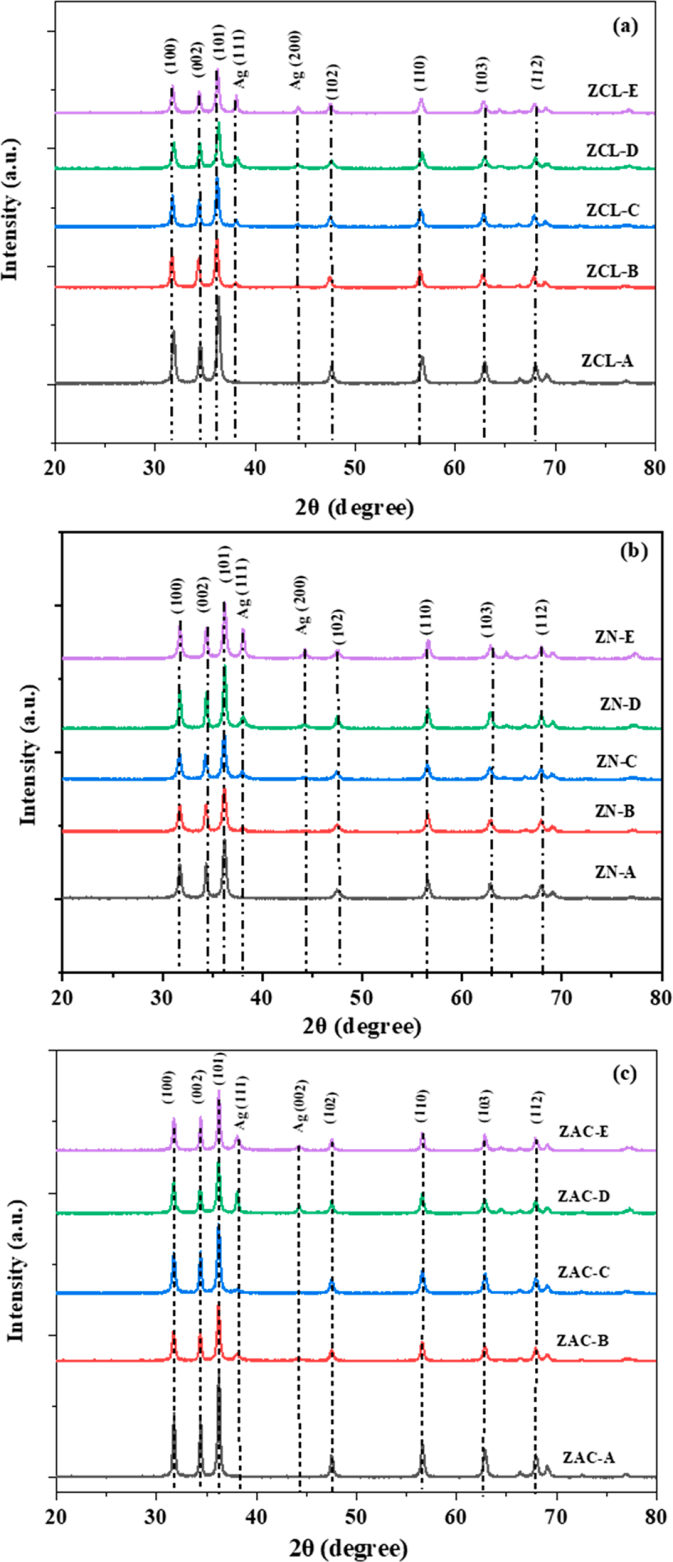
XRD pattern of (0, 2, 5, 7, and 10 wt %) Ag doped ZnO nanoparticles prepared using (a) ZCL, (b) ZN, and (c) ZAC precursors.

Figure. 1 shows XRD pattern of (0, 2, 5, 7, 10 wt %) Ag doped ZnO synthesized using ZCL, ZN, and ZAC. The peaks were observed to be broadened in XRD pattern confirming formation of nanocrystals in the array.^43^ Pristine ZnO has shown diffraction peaks confirming the presence of (100), (002), (101), (102), (110), (103), (200), and (112) planes for all zinc precursor samples showing the hexagonal phase (JCPDS Card No. 89-0511 and 80-0074).^44^ As we have doped silver into ZnO, it shows secondary phase formation corresponding to (111) and (200) planes (JCPDS card no 040783) having an FCC lattice.

In the Ag doped ZnO XRD pattern we have witnessed that the position of the (002) and (101) peaks experiences a slight alteration in the direction of smaller angles. Also with an increase in silver doping, the intensity of the diffraction peaks drops with peak widening.^45^ This leads to reduction of the crystalline nature in the synthesized nanoparticles upon increasing the silver doping. The alteration in position of the favorably adapted crystal planes (002) and (101) advocates replacement of Zn sites by silver ions. This alteration of peaks is not proportional to the wt % of doped silver but may be due to the variance in ionic radii of zinc and silver, since the ionic radii of silver ions are larger than those of zinc ions.^46^ The exchange of Ag with Zn sites was hard because of a notable variance in the ionic radii of silver and zinc, and instead silver ions get entangled with the surface of ZnO as also observed from TEM analysis. The average crystallite size, microstrain, and dislocation density were measured using the Scherrer formula as mention in Table ST-1 (Supporting Information).^47^ These outcomes show that no additional element is present in the synthesized samples. It is also observed that with the enhancement of Ag doping to ZnO there is a change in average crystallite size from 27 to 20 nm. This dropping in average crystallite size is credited to the induced stresses experienced by edges and boundaries during the growth and formation process with the increasing addition of silver^48,49^ as shown in Table ST-1 (Supporting Information).

### 3.2 Optical Studies

**Figure 2:**
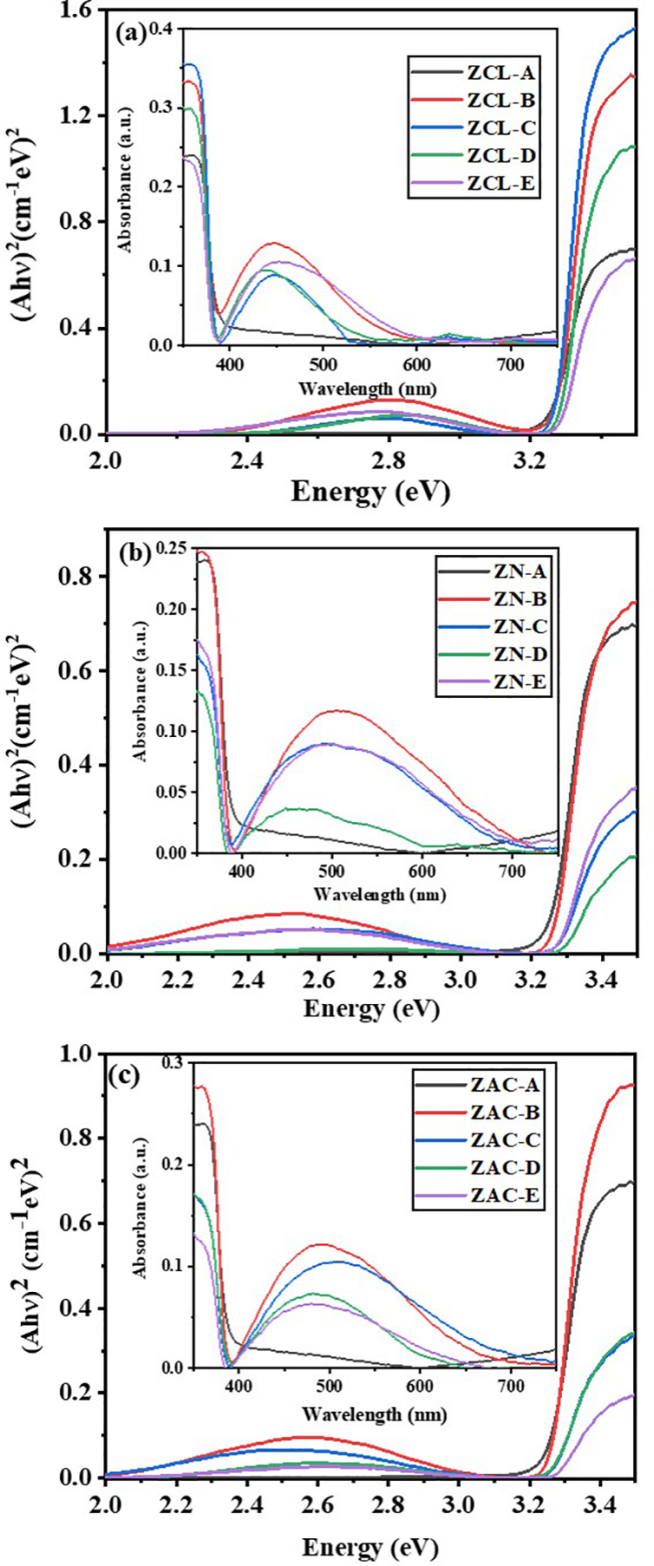
UV–vis spectra of (0, 2, 5, 7, and 10 wt %) Ag doped ZnO nanoparticles prepared using (a) ZCL, (b) ZN, and (c) ZAC precursors.

UV–visible absorption spectra for Ag doped ZnO samples prepared using ZCL, ZN, and ZAC precursor is shown in Figure 2(a), 2(b), and 2(c), respectively, showing a strong absorption edge in the UV region (200–420 nm) for ZnO.^43^ A plot of (*Ah*ν)^2^ versus photon energy (*hν*) is used to calculated band gap energy (*E*_g_) for pure and Ag doped ZnO samples, which was observed to be ∼3.2 eV with no significant change after doping. It is also witnessed that the gathering of Ag ions causes substantial alterations in the absorption spectrum of ZnO, showing the surface plasmon band as an extensive bulge in the visible region causing a large absorbance over the entire visible region with elevated Ag concentration.^34,45^ The surface plasmon scattering may be triggering the surge in the luminescence.^50^ Also the localized electric field associated at the junction of ZnO and Ag metal ions gets amplified causing excitation of surface plasmon in silver nanoparticles due to the increase in the absorption of incident visible irradiation,^51,52^ and density of accumulated silver nanoparticles on the exteriors of ZnO.

**Figure 3:**
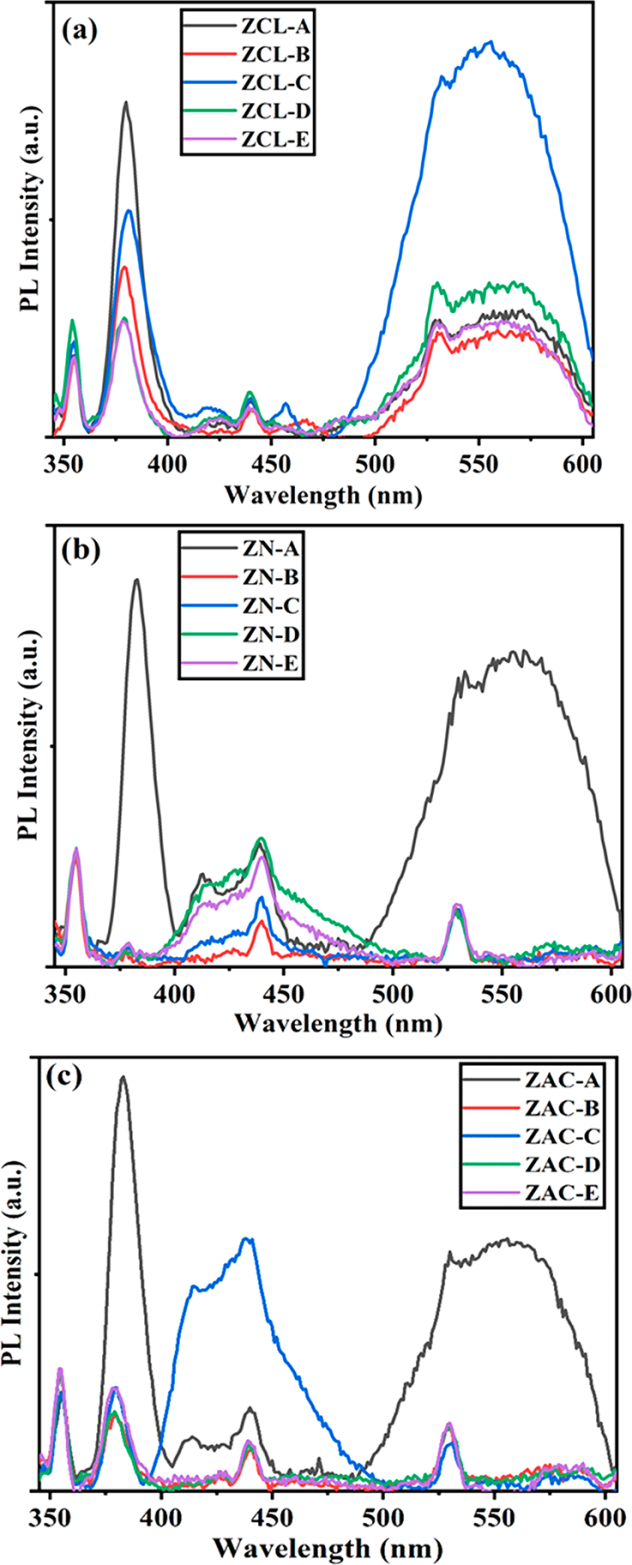
PL spectra of (0, 2, 5, 7, and 10 wt %) Ag doped ZnO nanoparticles prepared using (a) ZCL, (b) ZN, and (c) ZAC precursors.

PL spectroscopy is a generalized method to study the recombination of photogenerated charge carriers in a semiconductor.^53^ The recombination of charge carriers simultaneously with radiation gives rise to PL spectra where low PL intensity shows a low recombination rate.^53,54^Figure 3 shows the room-temperature PL spectra measured with an excitation wavelength of 320 nm for all Ag doped ZnO samples. A weak peak at 354 nm and prominent peak at 380 nm in the UV region is observed along with two frail bands in the range of 400 to 450 nm. A broader emission band is seen in the visible region positioned at 556 nm due to the radiative recombination of displaced electrons with holes in the oxygen interstitials (O_i_) located at 2.23 eV below the conduction band.^55,56^ The emission characteristics of pure and Ag doped ZnO samples are very much comparable, except with an supplementary peak at around 530 nm from the shift of electrons from Zn_i_ level located below the conduction band to the valence band.^50^ The intensity of emission drops with escalation in Ag doping concentration, signifying a quenching of PL emission, is also witnessed.^50,32^ This decrease in intensity of visible emission in silver doped catalysts may be attributed the plasmonic absorption of Ag nanoparticles, which is in good agreement with UV–vis spectra of the catalysts, where the Ag doped ZnO catalyst shows good absorbance near the 500 nm region in the UV–vis spectra.^55−57^ The sample prepared using zinc nitrate and zinc acetate precursors agrees more with above discussion as presented in Figure 3. The UV emission in the PL spectra may be caused by the wide band gap of ZnO. And the two weak bands in the visible region may be occurring due to imperfections present on the exteriors of the ZnO nanoparticles and bound electron hole pairs. This supports to a fact that due to incorporation of Ag nanoparticles there is a surge in the lifespan of the photogenerated charge carriers and increase in photocatalytic activity because of reduced surface defects in the Ag doped ZnO nanostructures.

### 3.3 Morphological Properties

**Figure 4:**
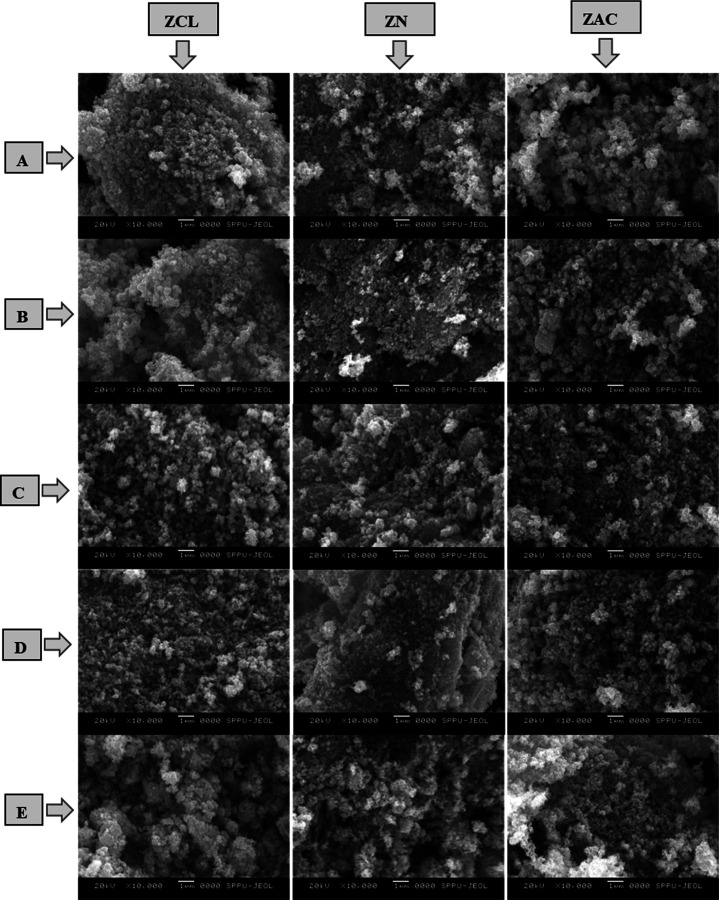
SEM micrograph of (0, 2, 5, 7, and 10) wt % Ag doped ZnO (A, B, C, D, and E, respectively) samples prepared using ZCL, ZN, and ZAC precursors at 10 K magnification.

Figure 4 displays the SEM micrograph of (0, 2, 5, 7, and 10) wt % Ag doped ZnO samples prepared using ZCL, ZN, and ZAC precursors at 10 K magnification. Agglomeration of particles and nonuniformity in particle sizes are observed from the SEM micrographs. As we increase the Ag doping concentration, there is an increase in agglomeration which can be due to the formation of AgO nanoparticles,^43,58^ and also the particle sizes differ with the increase in Ag doping concentration. Each SEM micrograph shows the presence of nanoflowers, with an average particle size of 25 to 30 nm calculated using ImageJ, which is in good agreement with XRD and TEM analysis. The bigger size particles are found because of agglomeration.

**Figure 5:**
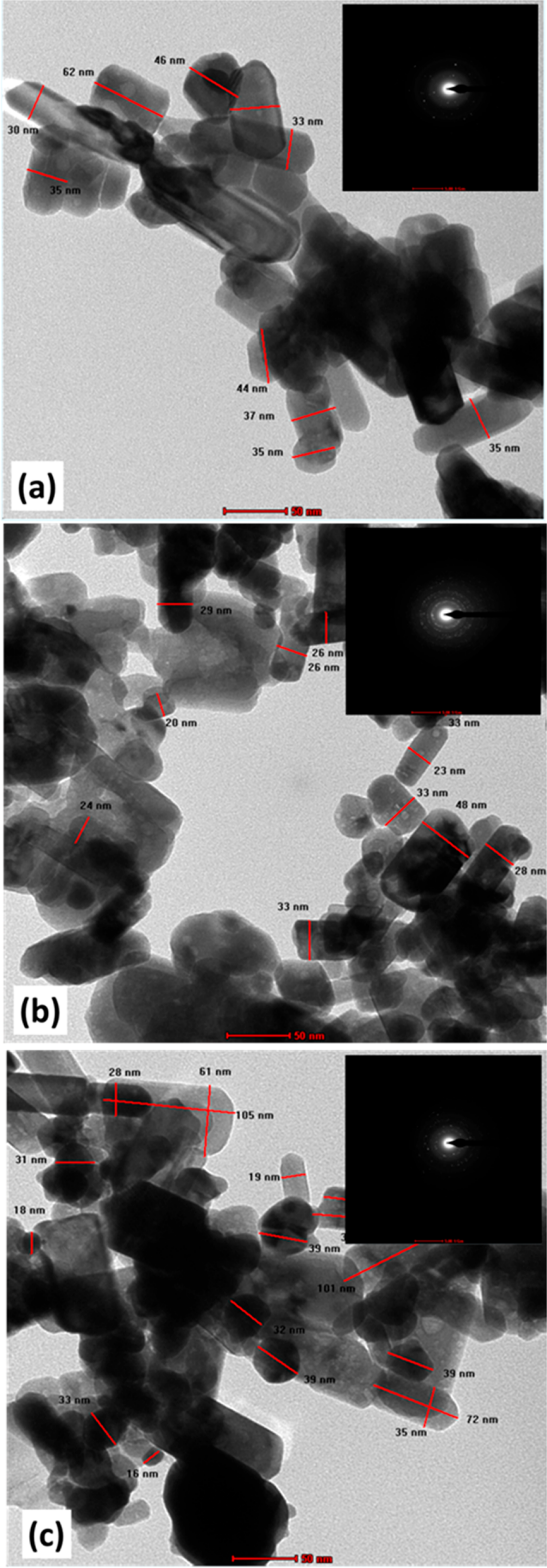
TEM micrograph of (a) 0, (b) 5, and (c) 10 wt % Ag doped ZnO samples prepared using ZAC precursors with SAED pattern inset.

To understand the effect of doping concentration TEM analysis was performed. Figure 5 shows TEM images of (0, 5, and 10 wt %) ZAC samples. ZnO nanoparticles show a cylindrical rod-like structure with a spherical agglomeration of doped Ag particles. Agglomeration occurs almost along all crystallite shape. ImageJ software was used to analyze and calculate the particle size of all samples. The average particle size calculated from TEM analysis is approximately 38 nm which is in good agreement with the average crystallite size calculated by XRD as shown in Table 1. The existence of Ag nanoparticles onto ZnO array can also be observed from TEM images, indicating reduction in particle size with an increase in doping concentration.^58^ This alteration in sizes of ZnO nanoparticles with Ag doping indicates that Ag bunches act as a nucleation site. Also an annular pattern is seen in SAED images which affirm the crystalline nature of the samples.

**Table 1:** Average Crystallite Size from XRD and TEM Analysis for ZAC Samples

Ag doping wt %	average crystallite size from XRD (nm)	average particle size from TEM (nm)
0	29	37
5	24	35
10	26	35

### 3.4 Compositional Properties

The energy dispersive spectroscopy (EDS) mapping probed the configuration and distribution of atoms present in Ag doped ZnO nanoparticles. The EDS mapping of doped samples show the even distribution of Ag metal in the ZnO array. Figure S-1 (Supporting Information) shows EDS spectrum of all samples prepared using ZCL, ZN, and ZAC precursors. The distribution of zinc (Zn) and oxygen (O) peaks in the matrix agrees with pureness of ZnO nanoparticles. The presence of Ag peak in the EDS array confirms the doping of silver in the ZnO. Table ST-2 (Supporting Information) shows the atomic wt % (0, 2, 5, 7, and 10) of Ag doped ZnO of all samples.^59^ Another observation recorded is that atomic weight percentage in catalysts prepared via zinc chloride precursor is lower than the actual weights used during the synthesis process, which may be caused by a partial amalgamation of silver which was prominently observed for catalysts prepared using zinc chloride precursor.

### 3.5 Photocatalytic Analysis

The solar light compelled photocatalytic activity of Ag doped ZnO nanoparticles was evaluated with MB and RB dyes as demonstrative pollutants. In this photocatalytic experiment, the dye solution was arranged by dissolving 10 mg MB dye in 1 L DDW. Five beakers, each contained 100 mL as the prepared dye solution was added with 0.1 g of (0, 2, 5, 7, and 10 wt %) Ag doped ZnO catalysts, separately. Further these beakers comprising the dye adsorbed photocatalyst were held under a visible light source of intensity 1 sun to perform the dye degradation experiment. Then 2 mL of dye solution was taken in intervals of 10 min as a sample followed by centrifuging and recording the UV–vis absorption spectra of the supernatant solutions.^60^ The experiments were repeated for 10 ppm RB dye solutions prepared in double distilled water for all catalyst samples. During the experimental process, 2 mL of dye solution was taken out as sample in the intervals of 5 min followed by centrifuging and recording UV–vis absorption spectra of the supernatant solutions.

**Figure 6:**
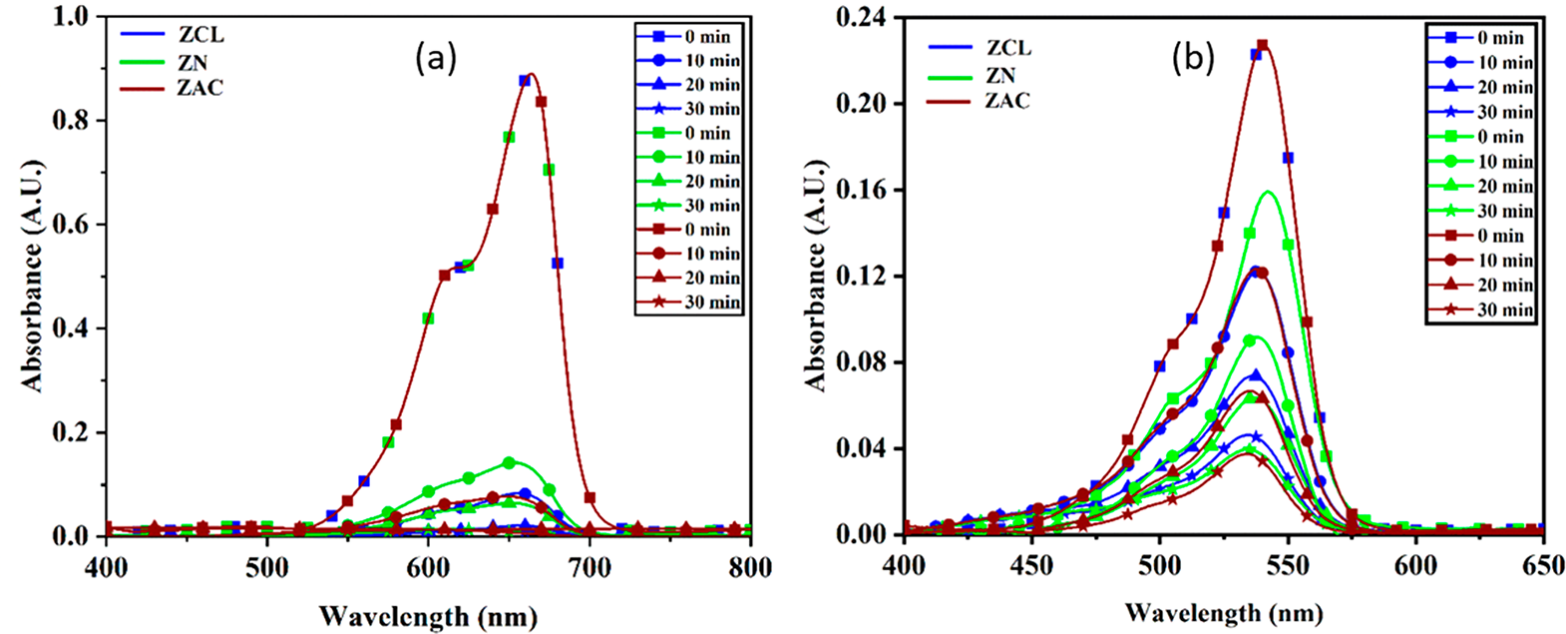
Photocatalytic degradation of (a) methylene blue and (b) rose bengal dye for 10 wt % Ag doped ZnO nanoparticles prepared using ZCL, ZN, and ZAC precursor.

**Figure 7:**
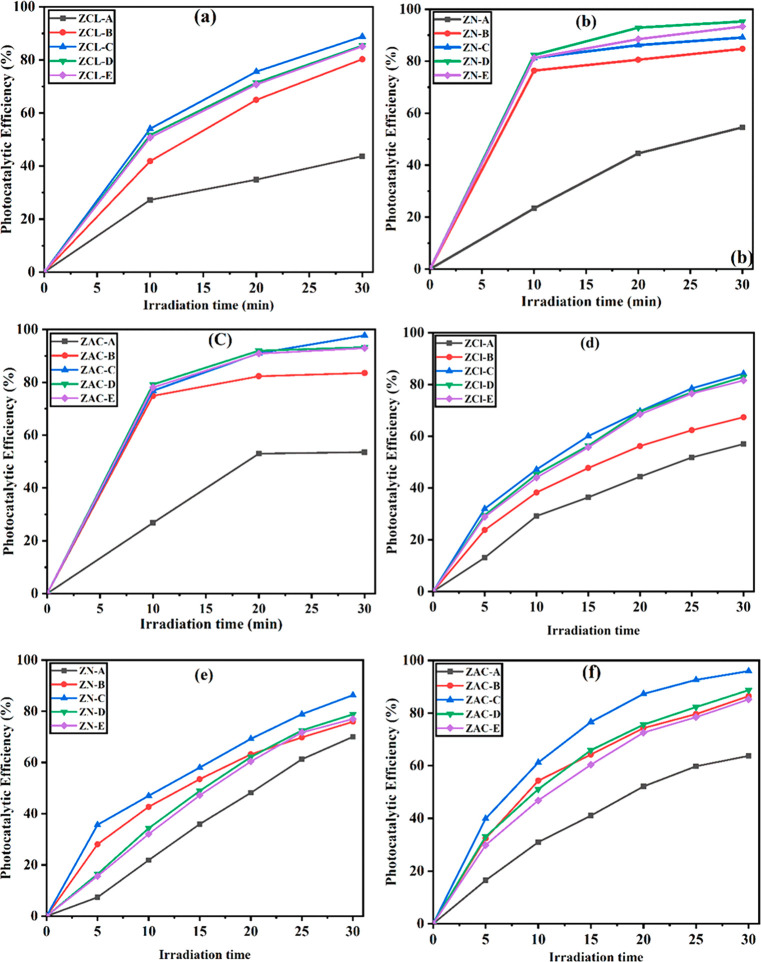
Plot of photocatalytic efficiency versus irradiation time of (0, 2, 5, 7, and 10 wt %) Ag doped ZnO nanoparticles for (a,b,c) methylene blue and (d,e,f) rose bengal using ZCL, ZN, and ZAC precursors, respectively.

Figure 7 represents comparative photocatalytic degradation of MB and RB dyes for 10 wt % Ag doped ZnO prepared using ZCL, ZN, and ZAC precursors, respectively. Figure S-4 (Supporting Information) represents comparative photocatalytic degradation of MB and RB dyes for (0, 2, 5, and 7) wt % of Ag doped ZnO nanoparticles. The degradation efficiency is measured using Beer–Lambert law, as shown in eq 1.^61,62^
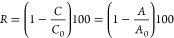
1where [*C*_0_, *A*_0_] and [*C*, *A*] represent concentration and absorbance of dye at reaction state time (0) and (*t*) minutes, correspondingly. The percentage dye degradation of all samples is measured using eq 1. Table 2 summarizes the rate constant and half-life for all samples. Absorption spectra show that the intensity of MB and RB dyes reduces with time upon exposure to visible light as shown in Figure S-4 (Supporting Information) and Figure 6.

**Table 2:** Rate Constant, Half Time, Photocatalytic Efficiency of Methylene Blue (MB) and Rose Bengal (RB) Dye Degradation (under 30 min Irradiation of Visible Light) and Antifungal Activity

sample name	rate constant (*x* 10^–2^ min^–1^)		half life (min)		photocatalytic efficiency (%)		% antifungal activity
	MB	RB	MB	RB	MB	RB	Bipolaris sorokiniana
ZCL-A	2	3	31	23	51	57	8
ZCL-B	5	4	13	16	80	67	17
ZCL-C	8	6	9	11	88	84	38
ZCL-D	7	6	10	12	85	83	45
ZCL-E	7	6	11	12	85	82	42
ZN-A	3	3	25	23	54	70	4
ZN-B	10	5	7	13	85	76	29
ZN-C	11	7	6	11	89	86	34
ZN-D	13	6	5	12	95	84	36
ZN-E	12	6	6	12	93	83	32
ZAC-A	3	4	23	19	54	64	7
ZAC-B	10	7	8	10	84	86	21
ZAC-C	13	10	6	7	98	96	32
ZAC-D	12	7	6	10	95	89	38
ZAC-E	12	6	6	11	93	85	35

It is observed that visible light absorption increases by doping of Ag and adds to efficient transmission of photogenerated charge carriers from agitated dye particle to the surface of ZnO via silver nanoparticles. Here Ag^+^ doped in ZnO arrests the photo induced electron in order to decrease the recombination of charges. The dye degradation activity was similarly carried in the dark condition for studying the outcome of adsorption. Both dyes had shown no noticeable influence of adsorption in the photocatalytic process. To explain the photocatalytic activity of given samples with respect to time, we have used the following kinetic model^63^
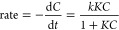
2where *C* is the concentration of dye (mg/L) at an instant ‘*t*’, ‘*t*’ is the time for which irradiation of sample takes place, *k* is first order constant of the reaction, and *K* is adsorption constant of dye on nanoparticles. Moreover this equation is simplified to the pseudo-first-order-equation.^64^
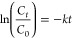
3Also, calculation of the half-life, *t*_1/2_, is performed using eq 4.^44^ All the data are tabulated in Table 2.
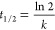
4

Figure S-2 (Supporting Information) shows the plot of relative concentration versus irradiation time, Figure S-3 (Supporting Information) shows plots of −ln (*C*/*C*_o_) versus irradiation time, and Figure 7 shows plots of photocatalytic efficiency versus irradiation time of (0, 2, 5, 7, and 10 wt %) Ag doped ZnO nanoparticles against MB and RB dye prepared using ZCL, ZN, and ZAC precursors, respectively.

**Figure 8:**
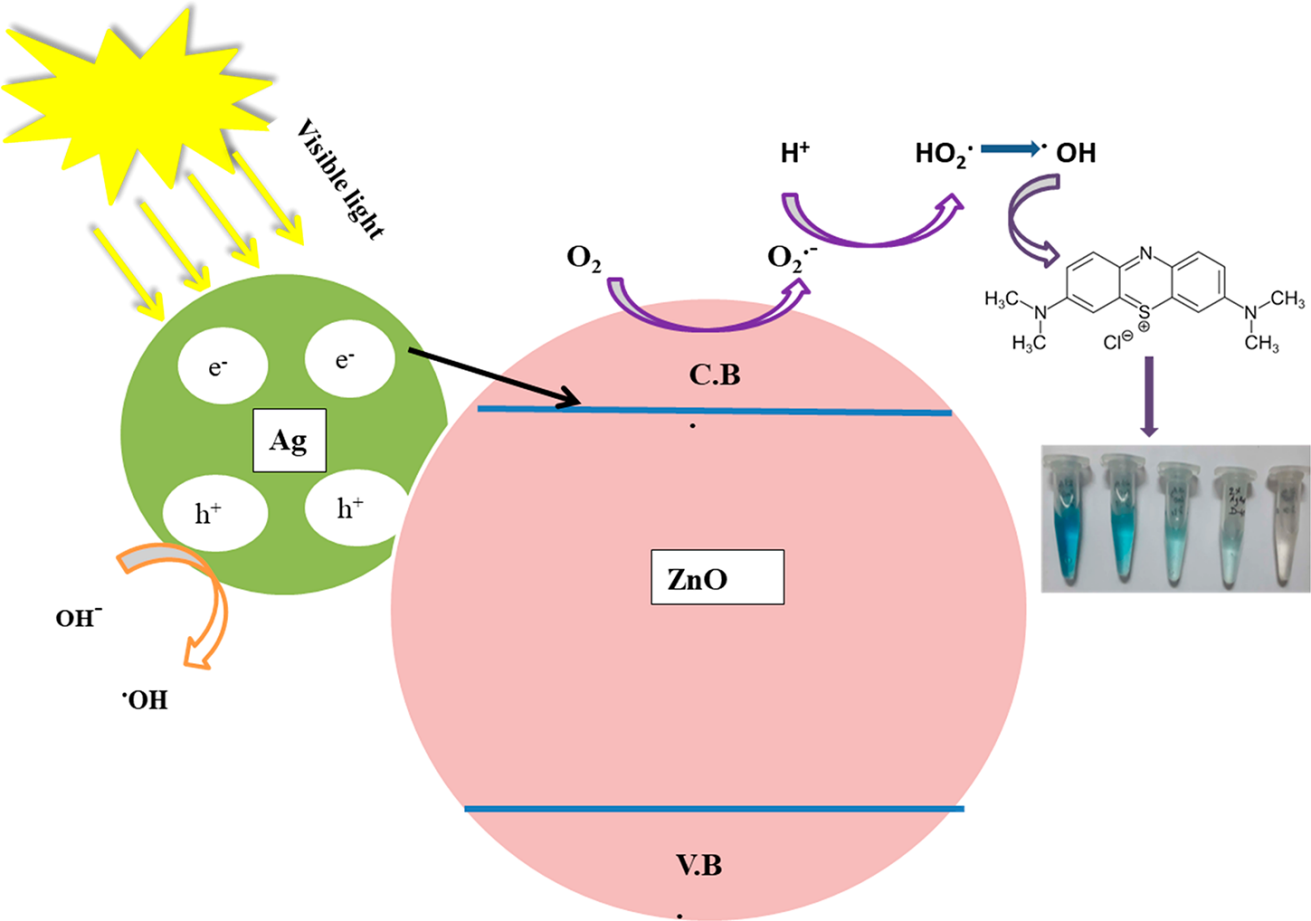
Working principle of methylene blue dye over Ag doped ZnO under irradiation of visible light.

The schematic of photocatalytic degradation process is shown in Figure 8.^52^ The surface plasmon resonance phenomenon generates the electron–hole pairs (equation 5). Thus, the plasmon-induced electrons of silver nanoparticles get rooted into the conduction band of ZnO (equation 6).^44,65^ This electron along with Ag trapped holes reacts with the preadsorbed oxygen (equation 7) and hydroxyl groups (^−^OH) present on the surface (equation 8), respectively, producing hydroxyl radicals. The produced OH radicals are adequate to disrupt the various bonds existing in the dye and lead to disintegration of the dye into CO_2_ and other ions.^66^ In due course, the OH radicals proceed to degrade the MB dye by hydrogen abstraction and subsequent oxidation processes (equation 9).^67−69^
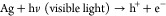
5
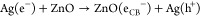
6
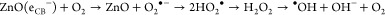
7
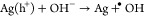
8
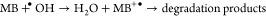
9

It is witnessed that, as the Ag dopant concentration rises, the photocatalytic efficiency initially rises and then slightly decreases as summarized in Table 2. There is significant enhancement in the degradation performance with silver doping in environmentally friendly and cost-effective ways. In previously reported literature the degradation efficiencies varied from 80 min to 4 h.^70^ From overall observation, the ZCL-C, ZN-C, and ZAC-C catalysts show optimum results of 88%, 89%, and 98% for annihilation of MB and 84%, 86%, and 96% for annihilation of RB under visible light irradiation of 30 min, which is an enhanced outcome as compared to our previously reported work.^41^

### 3.6 Antifungal Activity

In the present study, antifungal activity was checked on phytopathogenic fungi. Considering the antifungal activity of the silver nanoparticle, it is one of the potential applications for managing plant disease in the future compared to synthetic fungicides. Silver nanoparticles mainly affect the cell wall, surface protein, and nucleic acid of fungi by producing and accumulating ROS and free radicals.^71^ They also block the proton pumps.^72^ Antifungal activity was calculated by the poisoned food method.
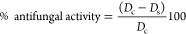
where *D*_c_ is the diameter of growth in the control plate, and *D*_s_ is the diameter in the plate containing silver nanoparticles. % antifungal activity of the silver nanoparticle is summarized in Table 1. Figure S-5 (supplementary data) shows inhibition of fungus against 7 wt % Ag doped ZnO.

## 4 Conclusions

In our current efforts, Ag doped ZnO nanoparticles were synthesized by the refluxed chemical method. From the XRD and TEM analysis, the average crystallite size and particle size are 27 and 35 nm, respectively. The quenching effect is observed in PL spectroscopy confirming the effect of silver doping in the photocatalyst. The photocatalytic activity against MB and RB dyes showed optimum degradation efficiency of 98% and 96% in 30 min, respectively, using 5 wt % Ag doped ZnO catalyst. Thus reduction in the energy band gap due to incorporation of silver doping into the ZnO array enhances the photocatalytic responses. Here we are first time reporting the usage of Ag doped ZnO against Bipolaris sorokiniana for which 7 wt % Ag doped ZnO has shown 45% inhibition efficiency. Thus the combined effects of surface defects, change in average crystallite size, different ionic radii of zinc and silver, SPR effect, and variation in doping concentration, are emphasized in this study, which contributes to improved annihilation of MB and RB dyes under visible light irradiation and antifungal activity against Bipolaris sorokiniana.

## Supporting Information Available

Parameters calculated from XRD analysis; elemental mapping of (0, 2 5, 7, and 10) wt % of Ag doped ZnO nanoparticles prepared using ZCL, ZN, and ZAC precursors; plot of relative concentration versus irradiation time and plots of −ln(*C*/*C*_o_) versus irradiation; photocatalytic degradation analysis for methylene blue and rose bengal dye; antifungal analysis (PDF)
