# Supplementary material for: Comparative Studies on Synthesis, Characterization and Photocatalytic Activity of Ag Doped ZnO Nanoparticles
Source: ACS Omega. 2023 Feb 13;8(8):7779–90. doi: 10.1021/acsomega.2c07499 (PMC9979246; doi:10.1021/acsomega.2c07499)
Supplement: Supplementary file 1 — ao2c07499_si_001.pdf [file ao2c07499_si_001.pdf]

Supporting Information

Comparative studies on synthesis, characterization and photocatalytic activity of Ag doped ZnO nanoparticles

Snehal S. Wagh[1,2,3], Vishal S. Kadam[2], Chaitali V. Jagtap[2], Dipak B Salunkhe[4] , Rajendra S. Patil[3,*], Habib M. Pathan,[2,5] and Shashikant P. Patole[5,*]

[1]School of Polytechnic and Skill development, Dr. Vishwanath Karad MIT World Peace University, Pune, 411038, India

[2]Advanced Physics Laboratory, Department of Physics, Savitribai Phule Pune University, Pune, 411007, India

[3]PSGVPM ASC College, Shahada, Nandurbar, 425409, India

[4]Kisan ASC College, Parola, Jalgaon, 425111, India

[5]Department of Physics, Khalifa University of Science and Technology, Abu Dhabi 127788, United Arab Emirates

Table ST-1. Parameters calculated from XRD analysis

Parameter	Crystal structure	Crystal size (D) nm	Micro strain (ε) (x 10^{-3})	Dislocation density 'δ' (x 10^{-3})
**ZCL-A**	Hexagonal	24	8.74	1.75
**ZCL-B**	Hexagonal	26	8.69	1.50
**ZCL-C**	Hexagonal	27	8.38	1.43
**ZCL-D**	Hexagonal	20	11.55	2.85
**ZCL-E**	Hexagonal	28	8.30	1.39
**ZN-A**	Hexagonal	23	9.69	2.22
**ZN-B**	Hexagonal	21	11.4	2.80
**ZN-C**	Hexagonal	20	12.6	2.92
**ZN-D**	Hexagonal	25	9.86	2.07
**ZN-E**	Hexagonal	24	9.61	2.07
**ZAC-A**	Hexagonal	30	7.02	1.19
**ZAC-B**	Hexagonal	25	9.44	1.82
**ZAC-C**	Hexagonal	25	10.73	2.66
**ZAC-D**	Hexagonal	26	8.57	1.51
**ZAC-E**	Hexagonal	27	9.19	1.90

Compositional Studies:

The EDS mapping of doped samples show the even distribution of Ag metal in ZnO array. Figure S-1 (a) shows EDS spectrum of ZCL-A, ZCL-B, ZCL-C, ZCL-D and ZCL-E. Figure. S-1 (b) shows EDS spectrum of ZN-A, ZN-B, ZN-C, ZN-D and ZN-E. Figure. S-1 (c) shows EDS spectrum of ZAC-A, ZAC-B, ZAC-C, ZAC-D and ZAC-E. The distribution of zinc (Zn) and oxygen (O) peaks in the matrix agrees the purity of ZnO nanoparticles. The existence of silver peak in Ag doped ZnO array contributes to the doping of silver in ZnO matrix.

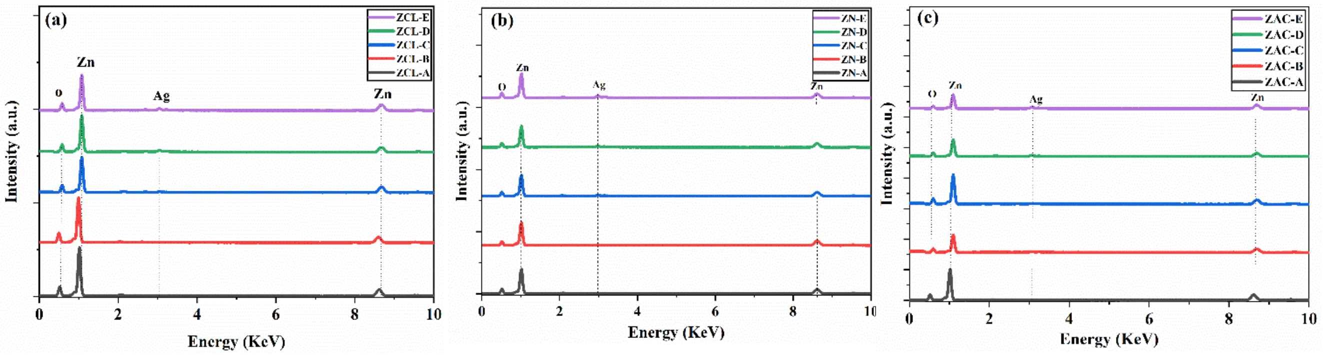

Figure S-1: EDS spectra of (0, 2, 5, 7, and 10 wt%) Ag doped ZnO nanoparticles prepared using (a) ZCL, (b) ZN, and (c) ZAC precursors.

Table ST-2: Wt. % of Ag doped ZnO prepared using ZCL, ZN and ZAC precursors.

Sample/ wt. %	Zn	O	Ag
**ZCL-A**	54	46	0
**ZCL-B**	57	40	2
**ZCL-C**	56	41	3
**ZCL-D**	49	45	6
**ZCL-E**	53	40	7
**ZN-A**	62	38	0
**ZN-B**	64	34	2
**ZN-C**	58	38	4
**ZN-D**	55	39	6
**ZN-E**	50	41	9
**ZAC-A**	62	38	0
**ZAC-B**	57	41	2
**ZAC-C**	57	38	5
**ZAC-D**	53	40	7
**ZAC-E**	57	33	10

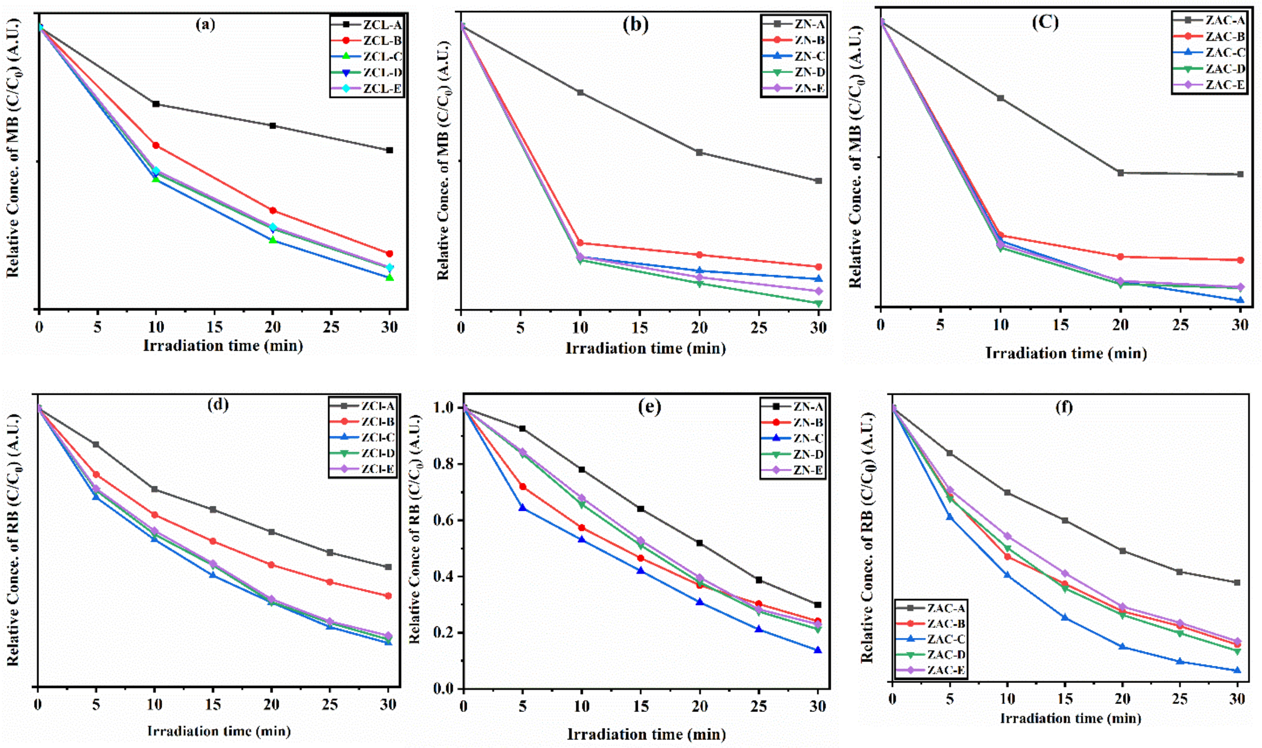

Figure S-2: Plot of relative concentration versus irradiation time of (0, 2, 5, 7 and 10 wt%) Ag doped ZnO nanoparticles for (a), (b), (c) methylene blue and (d), (e), (f) rose bengal using ZCL, ZN and ZAC precursors respectively.

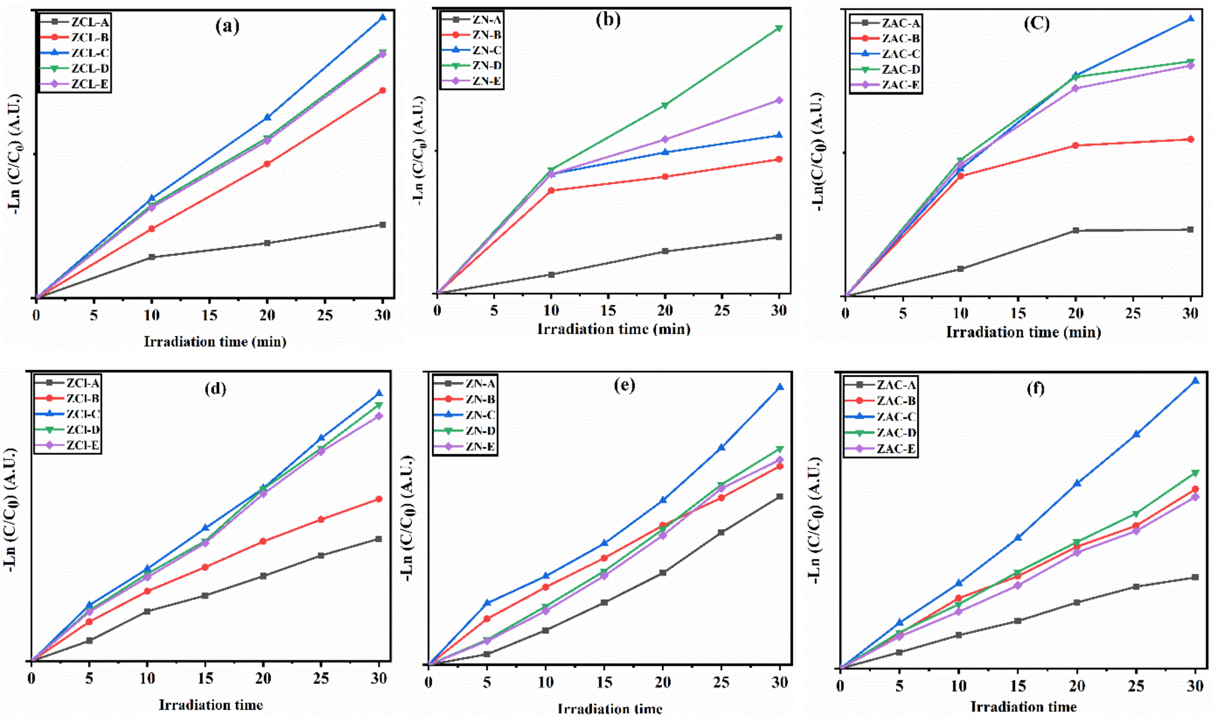

Figure S-3: Plots of - ln(C/C_o) versus irradiation time of (0, 2, 5, 7, and 10 wt%) Ag doped ZnO nanoparticles for (a), (b), (c) methylene blue and (d), (e), (f) rose bengal using ZCL, ZN, and ZAC precursors respectively.

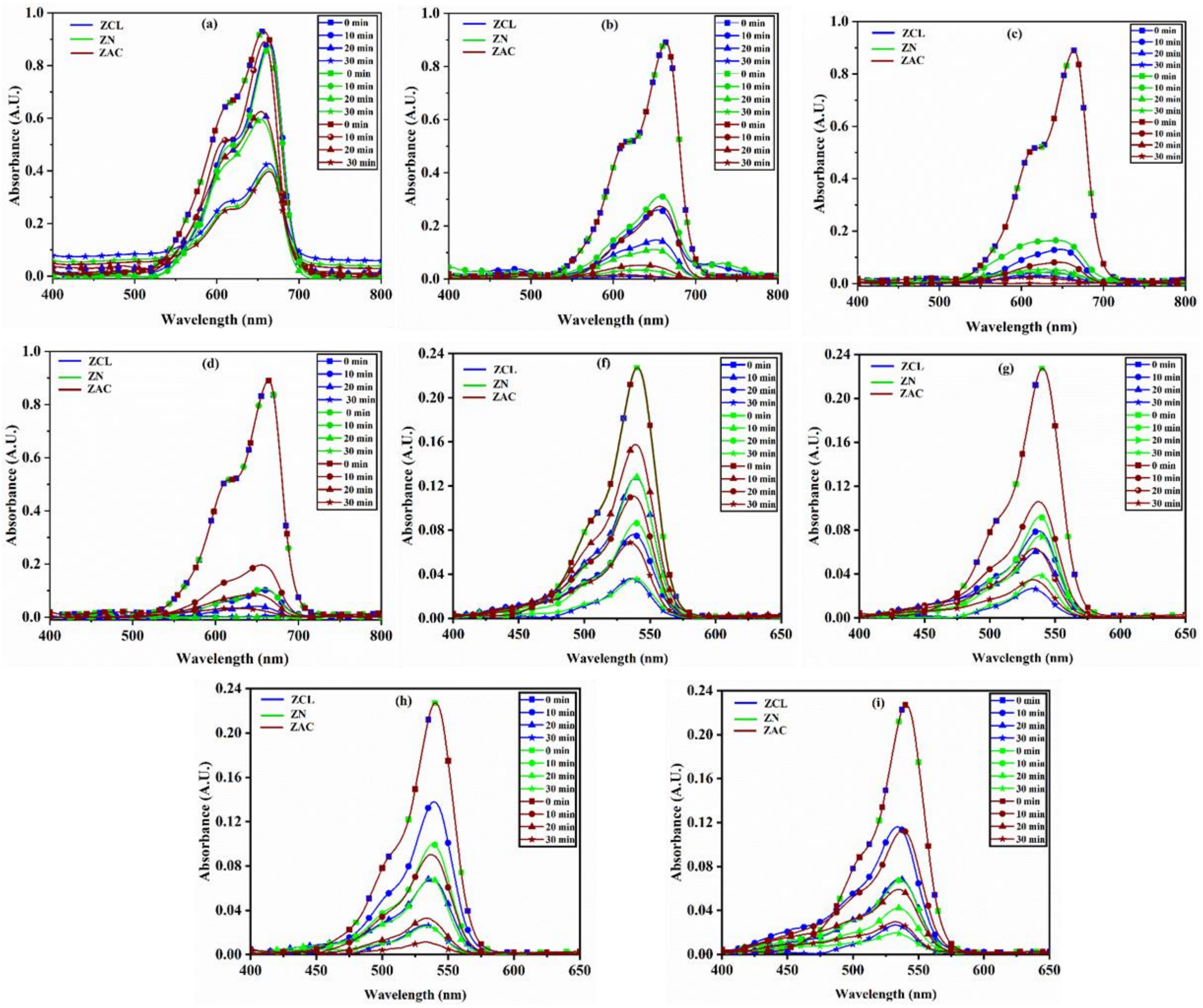

Figure S-4: Photocatalytic degradation of (a), (b), (c), (d) methylene blue and (f), (g), (h), (i) rose bengal dye for (0, 2, 5, and 7 wt.%) Ag doped ZnO nanoparticles prepared using ZCL, ZN, and ZAC precursor.

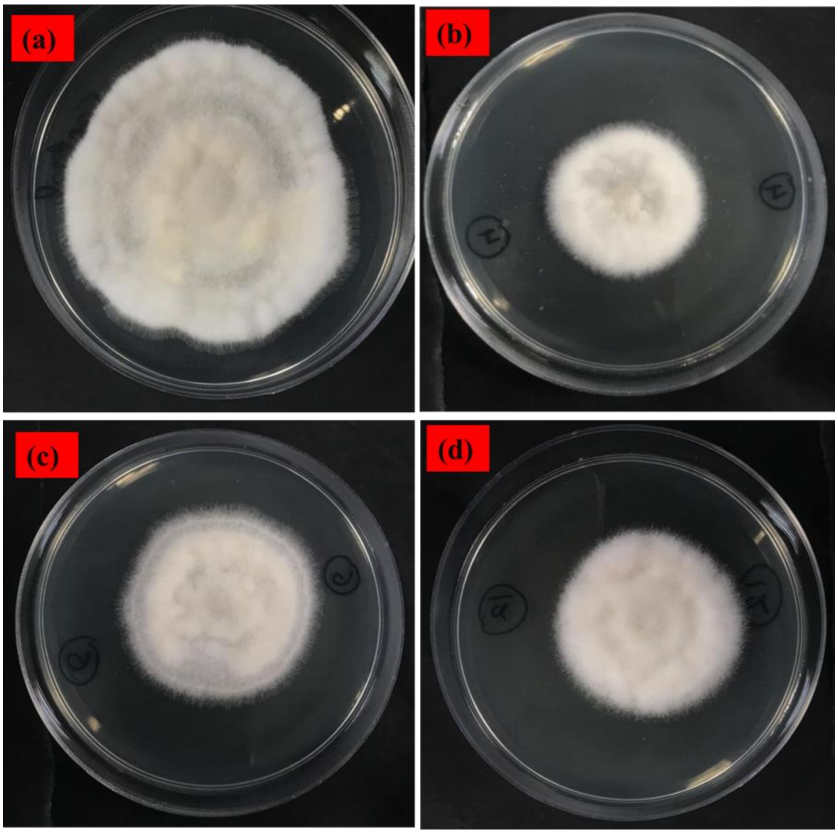

Figure S-5: Antifungal activity of 7 wt.% Ag doped ZnO against Bipolaris sorokiniana. Effect of different concentrations on the antifungal potency (a) control, (b) ZCL-D, (c) ZN-D, and (d) ZAC-D.
